# Preoperative anxiety in adults - a cross-sectional study on specific fears and risk factors

**DOI:** 10.1186/s12888-020-02552-w

**Published:** 2020-03-30

**Authors:** Leopold Eberhart, Hansjörg Aust, Maike Schuster, Theresa Sturm, Markus Gehling, Frank Euteneuer, Dirk Rüsch

**Affiliations:** 1 Philipps-University Marburg, Biegenstraße 10, 35037 Marburg, Germany; 2 Department of Anesthesia and Intensive Care, University Hospital Giessen-Marburg, Marburg Campus, Baldingerstraße, 35033 Marburg, Germany; 3 Department of Psychology, Medical School Berlin, Berlin, Germany; 4 Division of Clinical Psychology and Psychotherapy, Philipps-University Marburg, Marburg, Germany

**Keywords:** Cross-sectional survey, Preoperative anxiety, Anesthesia related anxiety, Surgery related anxiety, Specific concerns, Independent predictors, Multivariate regression

## Abstract

**Background:**

Preoperative anxiety comprising anesthesia and surgery related anxiety is common and perceived by many patients as the worst aspect of the surgical episode. The aim of this study was to identify independent predictors of these three anxieties dimensions and to quantify the relevance of specific fears particularly associated with anesthesia.

**Methods:**

This study was part of a cross-sectional survey in patients scheduled to undergo elective surgery. Anxiety levels were measured with the Amsterdam Preoperative Anxiety and Information Scale (APAIS). Modified numeric rating scales (mNRS, range 0–10) were used to assess the severity of eight selected specific fears which were predominantly analyzed descriptively. Multivariate stepwise linear regression was applied to determine independent predictors of all three anxiety dimensions (APAIS anxiety subscales).

**Results:**

3087 of the 3200 enrolled patients were analyzed. Mean (SD) total preoperative anxiety (APAIS-A-T, range 4–20) was 9.9 (3.6). High anxiety (APAIS-A-T > 10) was reported by 40.5% of subjects. Mean (SD) levels of concern regarding the eight studied specific fears ranged from 3.9 (3.08) concerning “Anesthesiologist error” to 2.4 (2.29) concerning “Fatigue and drowsiness” with an average of 3.2 (2.84) concerning all specific fears. Ranking of all specific fears according to mean mNRS scores was almost identical in patients with high versus those with low anxiety. Among nine independent predictors of anxiety, only 3 variables (female gender, negative and positive anesthetic experience) independently predicted all three APAIS anxiety subscales. Other variables had a selective impact on one or two APAIS anxiety subscales only. Female gender had the strongest impact on all three APAIS anxiety subscales. Adjusted r^2^ values of the three models were all below 13%.

**Conclusions:**

The high variability of importance assigned to all specific fears suggests an individualized approach is advisable when support of anxious patients is intended. Considering independent predictors of anxiety to estimate each patient’s anxiety level is of limited use given the very low predictive capacity of all three models. The clinical benefit of dividing patients into those with high and low anxiety is questionable.

**Trial registration:**

German Registry of Clinical Trials (DRKS00016725), retrospectively registered.

## Background

Preoperative anxiety is of relevance for anesthetists and surgeons. According to results of an observational study in more than 15,000 patients undergoing a non-obstetric surgical procedure, anxiety was most frequently mentioned to be the worst aspect of the perioperative period [1]. Preoperative anxiety includes anxiety about both anesthesia and surgery. The intensities of the latter differ significantly in many patients [2] suggesting that underlying specific fears and predictors of these two anxiety dimensions may be distinct. In order to help patients with their anesthesia related anxiety, it may be helpful to be familiar with the prevalence and above all the relevance of specific fears associated with anesthesia related anxiety from a patient perspective. This requires, when talking to patients that can be classified as monitors (2/3 of this sample of patients were shown to be monitors [3]) to also be familiar with the likelihood of the matter of concern and with the strategies implemented in the institution to prevent the various adverse event from happening (e.g. use of depth of anesthesia monitors to prevent intraoperative awareness). Both limited time available for patients to be seen preoperatively and above all the specialization in medicine suggests that anesthesiologist should focus on specific fears related to their own field (e.g. “awareness”) without ignoring general concerns (e.g. “nudeness” [4] and fears associated primarily with the surgical procedure (e.g. “result of operation” [5]). In addition, knowledge of the prevalence and importance of these concerns could be helpful for creating patient education materials. Various studies have examined anxiety provoking aspects underlying preoperative anxiety [4–12] with only some of these studies reporting intensities of concern [4, 5, 7, 9] and few of these focusing on potential adverse events and complications particularly associated with anesthesia [4, 6, 9]. To date, previous studies have failed to unanimously identify the most anxiety provoking aspects of anesthesia. This is most likely caused by the lack of standardization of studies examining specific fears and risk factors associated with preoperative anxiety. As a result, study designs differ in numerous ways that include for instance enrolled subjects, time and place of data acquisition, instruments used for data acquisition, and data analysis. One important aspect in this context is a limited overlap concerning the more than 60 items examined in the different surveys [4–13]. Another important limitation of past work is that none of the previous studies have measured mean intensities and the amount of variability of specific fears concerning anesthesia related anxiety. Differences in study designs make comparing results extremely difficult and also impede verification of previous results as this would require to integrate the different study designs in one study. Therefore, the main goal of this study was to reassess the relevance of selected anxieties associated with general anesthesia that had been examined in the majority of previous surveys [4–13] by measuring for the first time their mean intensities and the variability in a large sample of patients in order to identify the most anxiety provoking concerns with regard to anesthesia.

Knowledge of potential risk factors for preoperative anxiety also appears to be important in this context as it may be helpful in identifying those patients likely to experience elevated levels of preoperative anxiety. Many patient variables have been studied in the past regarding their association with preoperative anxiety. Among these, only the female gender has consistently been shown in numerous studies to be a risk factor for preoperative anxiety (e.g. [5, 14–20]). In contrast, inconsistent results have been published for various other patient characteristics including age [5, 21], education [5, 15, 17, 21], history of cancer [15, 21], previous surgery [5, 14, 17–19, 21, 22], and the grade of surgery as well as the surgical discipline [5, 15, 19, 21]. Among the many differences with regard to study design mentioned above which could account for these inconsistencies, differences in statistical analyses seem to be particularly important in this context. Little evidence exits regarding the association between preoperative anxiety and the kind of experience (positive or negative) patients have had with previous surgeries [5] and associations with mutilating surgery have not been studied at all. In addition, to date no previous study has assessed if predictors differ between anesthesia and surgery related anxiety. The secondary aim of this study therefore, was to examine the association between above mentioned variables and preoperative anxiety as well as for the first time anesthesia related anxiety and surgery related anxiety separately.

Because of time constraints in everyday clinical practice, Moerman and colleagues suggested to identify by means of a scoring system patients with high anxiety (“anxiety cases”) that may benefit from more attention and information [14]. Accordingly, many later studies examining preoperative anxiety applied a cut-off score using validated anxiety scoring tools to identify anxiety cases in order to be able to focus part of their research on these anxiety cases (e.g. [5, 19, 21, 23, 24]). Moerman and colleagues also proposed that future research should be conducted to clarify whether it is useful to distinguish between anxiety cases and nonanxiety cases. Accordingly, this aspect was included in the analysis concerning the two main objectives in order to evaluate for the first time the clinical usefulness of distinguishing between patients with high and low anxiety.

## Methods

This study was part of a cross-sectional survey conducted to explore various aspects concerning preoperative anxiety and coping with preoperative anxiety in the most comprehensive sample of patients studied so far in this respect. Results of the survey regarding strategies to cope with preoperative anxiety [3] and findings about prevalences and intensities of preoperative anxiety, anxiety about surgery and anxiety about anesthesia [2] have recently been published. This paper reports on results of the survey concerning (a) specific fears underlying preoperative anxiety and anxiety about anesthesia in particular and (b) predictors of total preoperative anxiety as well as predictors of anxiety about anesthesia and anxiety about surgery.

### Ethics approval and study registration

This survey was approved by the ethics committee of the Medical faculty of Marburg University (Approval number: 18/12, dated March 1st 2012). It was registered retrospectively at the German Registry of Clinical Trials (DRKS-ID: DRKS00016725).

### Study subjects

Adult patients who were scheduled to undergo any kind of surgical procedure under anesthesia were eligible to be enrolled in this survey. Exclusion criteria for this survey included illiteracy, insufficient knowledge of the German language, mental disorders and impaired visual acuity which would prevent patients from completing a questionnaire. In accordance with the decision of the local ethics committee, no written consent for participation was required because of the voluntary character of this anonymous survey. After information was provided to eligible patients about the methodology and aims of the survey, informed consent to participate was taken verbally, followed by completion of the questionnaire. Completion of the questionnaire could be stopped at any time without giving any reason.

### Data collection

This survey was conducted at the pre-anesthetic evaluation clinic of Marburg University Hospital in patients who were waiting for their preoperative face-to-face assessment with a physician of the Department of Anesthesia and Intensive Care. Following verbal consent, they were given the questionnaire and asked to complete it which took on average 10–15 min. A member of the study team was present to answer any questions the patients had.

### Questionnaire

The questionnaire used for this survey contained three sections (A: patient characteristics, B: anxiety, C: coping) which were specifically designed for this survey in cooperation with the Department of Clinical Psychology and Psychotherapy, Marburg University. Section A consisted of one questionnaire (A-1) which covered standard sociodemographic characteristics (age, gender, education), aspects related to the procedure (referring surgical discipline, kind of procedure, benign or malignant tumor), the number of previous surgeries, patients’ feelings concerning the intervention (i.e. whether the surgery was accompanied by a burdensome change of the body, sometimes referred to as mutilating or symbolically castrating surgery) as well as experiences with previous anesthetics (i.e. whether subjects, relatives or friends have had a positive or negative experience with a previous anesthetic). Section B included three parts (B1–3) about certain aspects of preoperative anxiety. Part B1 consisted of a German version (Additional file 1A) of the validated Amsterdam Preoperative Anxiety and Information Scale (APAIS) [14] (Additional file 1B) which contains six items (statements) to assess the magnitude of patient’s anesthesia related anxiety (APAIS-A-An, two items, score 2–10), surgery related anxiety (APAIS-A-Su, two items, score 2–10) and need for information (APAIS-I, two items, score 2–10). Total preoperative anxiety (APAIS-A-T, four items, score 4–20) is the sum of anesthesia and surgery related anxiety (APAIS-A-An plus APAIS-A-Su). The APAIS has previously been described in detail and validated in many countries using different languages [25–28] including a German version [29]. Recently published results of this survey demonstrated that the reliability (Cronbach’s α) of the four anxiety items (“anxiety scale”) and of the two information items (“information scale”) were 0.87 and 0.74, respectively [2]. Given the strong evidence concerning the validity and reliability of the APAIS it can be considered the gold standard to measure preoperative anxiety.

Part B2 comprised two rating scales to separately assess anxiety about anesthesia and anxiety about surgery. The rating scales used in this part of the questionnaire can be best described as a modified numeric rating scale (mNRS) ranging from 0 (no anxiety) to 10 (extreme anxiety) in horizontal orientation with a triangle above the numbers indicating increasing anxiety from left to right (Additional files 2A and B). This second instrument to assess anxiety levels was used to re-confirm results obtained with the APAIS. Results concerning the two parts of section B (parts B1 and B2) have recently been published [2].

Part B3 contained 8 items representing the most frequently examined typical adverse events and common fears associated with general anesthesia [4, 6, 9] among all specific fears associated with preoperative anxiety that have been studied so far [4–11, 13] . These 8 items included anxiety about painful measures, loss of control, waking up during surgery / intraoperative awareness, anesthesiologist error, not waking up / death under anesthesia, postoperative nausea and vomiting, fatigue and drowsiness, and permanent impairment of personality [Additional file 3A and B]. Patients were asked to rate their anxieties using the mNRS that ranges from 0 (no anxiety) to 10 (extreme anxiety).

### Additional information from medical records

The patients’ medical records were checked after return of the completed questionnaires to find out the type of surgery scheduled in case the corresponding section of the questionnaire had not been completed. If necessary, a surgeon was contacted in order to enable assignment of the procedure to one of the procedure types.

### Sample size

The sample size of the survey was primarily based on the sample size calculation from the study on coping with preoperative anxiety [3], described again in detail very recently [2]. Considering the results of that sample size calculation and in order to be able to compensate for drop-outs, 3200 patients were enrolled in this survey.

### Data processing and statistical analysis

All procedures were categorized into 10 groups (Additional file 4, Table 1) based on the anticipated mental burden of the procedure and the surgical invasiveness of the operation. To date, there is no uniform, internationally approved classification of surgical procedures that primarily addresses these two aspects. In addition, none of the classifications used in the literature discriminating between two to four grades of surgery only (e.g. [15, 21, 30] is sufficiently differentiated to reveal the impact of psychological aspects on intensities of preoperative anxiety. Therefore, we created a much more differentiated classification of surgical procedures including for the first time psychological aspects related to surgery. For instance, one group comprised surgical procedures aiming at pain reduction and/or improvement of function assuming that these procedures might be associated with a lower level of anxiety related to the procedure. On the other hand, another group included all procedures involving open surgery of the brain or of the heart assuming these procedures might be associated with higher levels of anxiety because of the extreme invasiveness of the procedure and the fact that the procedure involves an extremely important organ of the body.

**Table 1:** Type of procedures

Type of procedure	Subgroup	Typical example
1. Diagnostic procedure	1.1 Arthroscopy	Knee arthroscopy
	1.2 Diagnostic laparoscopy	Diagnostic pelviscopy in patients with endometriosis or fertility problems
	1.3 Endoscopies + excisional biopsy	Biopsy of mediastinal lymph nodes using endobronchial ultrasound (EBUS)
2. Medical imaging, radiation therapy, catheter placement, implantation of cardiac devices, ablative interventional radiological procedures	2.1 Magnetic resonance imaging (MRI)	MRI in patients with claustrophobia
	2.2 Brachytherapy or external beam radiation	Brachytherapy of prostate cancer
	2.3 Catheter placement	CAPD catheter, Port
	2.4 Pacemaker and ICD implantation	
	2.5 Radiofrequency ablation (RFA), cryoablation, chemoembolization (TACE)	RFA of hepatocellular carcinoma, cryotherapy in bone tumors, TACE in metastases of neuro-endocrine neoplasms
3. Trauma-related non-emergency surgery	3.1	Plate and screw osteo-synthesis for distal radius fracture, anterior cruciate ligament reconstruction
4. Surgery aimed at pain reduction and / or improvement of function	4.1 All interventions except for eye surgery	Total knee replacement, femoropopliteal bypass, septoplasty
	4.2 Eye surgery	Vitrectomy, cataract surgery
5. Resection (benign)	5.1	Thyroidectomy, transurethral resection of prostate, hysterectomy
6. Resection (malignant)	6.1	Breast conserving therapy, Abdominoperineal rectal resection, Radical prostatectomy
7. Highly invasive surgery	7.1 Neurosurgery involving craniotomy	Excision of meningioma
	7.2 Cardiac surgery involving sternotomy	Aortocoronary bypass, cardiac valve replacement
8. Cosmetic and reconstructive surgery	8.1	Rhinoplasty, breast reconstruction after mastectomy
9. Obstetrics	9.1	Elective cesarean section
10. Miscellaneous	10.1	Analgesic pump explantation, closure of tracheostomy stoma

Classification of all procedures based on the anticipated mental burden of the procedure and the surgical invasiveness of the operation

Incomplete questionnaires and questionnaires with contradictory answers were excluded from analysis. Data analysis was performed using JMP 14 (SAS Institute Inc., SAS Campus Drive, Cary, NC, USA 27513). Descriptive statistics include mean and standard deviation (SD), and / or median, 10 and 90% percentiles for continuous variables as well as numbers and percentages for discrete variables.

Mean intensities of each specific fear were compared between patients with high (APAIS-A-T > 10) and low (APAIS-A-T < 11) anxiety using Mann-Whitney-U-test. The ratio between the intensities of each specific anxiety in patients with high and low anxiety was calculated to examine whether any specific fears were of particular relevance in patients with high compared to patients with low anxiety. The incidence of patients with a mNRS score > 0 was calculated for each specific fear to determine the fraction of patients affected by these in all patients, patients with high and with low anxiety. Associations between intensities of all specific fears were studied by performing a factor analysis in order to identify underlying dimensions. The maximum-likelihood method was used for factor extraction. After varimax rotation, a model with three factors was found to be sufficient, explaining a cumulative variance of 67.5%.

The impact of independent variables (e.g. patient characteristics) on preoperative anxiety was studied in two ways. Firstly, 95% confidence intervals of prevalences of independent variables in patients with high anxiety versus patients with low anxiety were compared. Secondly, associations between patient characteristics, surgical discipline, type of surgery and previous experiences as independent variables and total anxiety score (APAIS-A-T), anxiety about surgery score (APAIS-A-Su) and anxiety about anesthesia score (APAIS-A-An) as dependent variables were analyzed using multivariate stepwise linear regression. Primarily a forward procedure was performed and stopped when Bayessian information criterion (BIC) had reached its minimum. To validate the three models a backward procedure was also calculated and alternative stopping rules (AIC or *p*-value) were applied. Adjusted r^2^-values were calculated to determine how well the variances of the three anxiety measures (APAIS anxiety subscales) were explained by the independent variables. Need for information was intentionally not included in the analysis because previously published results of this survey clearly demonstrated, that monitors and blunters among patients with high anxiety had almost identical total anxiety levels examined by using the APAIS (APAIS-A-T: 13.45 ± 2.2 vs. 13.50 ± 2.2) This was also the case when comparing monitors and blunters using a VAS (VAS-Anesthesia anxiety: 5.6 ± 2.4 vs. 5.6 ± 2.3; VAS-Surgery anxiety: 6.6 ± 2.1 vs. 6.5 vs. 2.2) [3].

## Results

Three thousand two hundred patients were enrolled from March 2012 until April 2013. After having excluded 113 questionnaires due to violation of inclusion criteria (4), contradictory answers (7) and incompleteness (102), data from 3087 patients could be analyzed. Participants (57% female) of this survey were 50 years on average. Other patient characteristics are presented in Table 2.

**Table 2:** Patient characteristics

	All patients	High anxiety	Low anxiety	APAIS-A-T* [M (SD)]	APAIS-A-An* [M (SD)]	APAIS-A-Su* [M (SD)]
Age (years) [M (SD)]	50 (17)	49 (17)	51 (17)			
All patients				9.9 (3.6)	4.3 (1.9)	5.5 (2.1)
Gender [n (%; 95% CI)]						
Female	1773 (57; 56–59)	891 (71; 69–74)	954 (48; 46–50)	10.7 (3.6)	4.8 (2.0)	6.0 (2.1)
Male	1314 (43; 41–44)	360 (29; 26–31)	882 (52; 50–54)	8.7 (3.2)	3.7 (1.6)	4.9 (2.0)
Education ^a^ [n (%; 95% CI)]						
≤ 9 years	1077 (35; 33–37)	443 (35; 33–38)	634 (35; 32–37)	9.8 (3.8)	4.3 (2.0)	5.5 (2.2)
10 years	1063 (34; 33–36)	448 (36; 33–39)	615 (34; 31–36)	10.0 (3.6)	4.4 (1.9)	5.6 (2.1)
≥ 13 years	947 (31; 29–32)	360 (29; 26–31)	587 (32; 30–34)	9.8 (3.4)	4.2 (1.8)	5.6 (2.0)
Anesthesia experience [n (%; 95% CI)]						
None	485 (16; 14–17)	189 (15; 13–17)	296 (16; 14–18)	9.7 (3.7)	4.3 (1.9)	5.5 (2.2)
Only good	1768 (57; 56–59)	618 (49; 47–52)	1150 (63; 60–65)	9.4 (3.5)	4.1 (1.8)	5.4 (2.1)
Only bad	138 (5; 4–5)	87 (7; 6–9)	51 (3; 2–4)	11.8 (3.9)	5.5 (2.2)	6.3 (2.3)
Good and bad	696 (22; 21–24)	357 (29; 26–31)	339 (18; 17–20)	10.7 (3.6)	4.8 (2.0)	5.9 (2.1)
Previous surgeries [n (%; 95% CI)]						
None	333 (11; 10–12)	157 (13; 11–15)	176 (10; 8–11)	10.5 (3.6)	4.7 (1.9)	5.8 (2.0)
1–2	1154 (37; 36–39)	484 (39; 36–41)	669 (36; 34–39)	10.0 (3.6)	4.4 (1.9)	5.6 (2.1)
≥ 2	1600 (52; 50–54)	610 (49; 46–52)	991 (54; 52–56)	9.6 (3.6)	4.2 (1.9)	5.5 (2.1)
Malignant tumor ^b^ [n (%; 95% CI)]	409 (13; 12–15)	184 (15; 13–17)	225 (12; 11–14)	10.5 (3.7)	4.4 (1.9)	6.1 (2.2)
Mutilating surgery ^c^ [n (%; 95% CI)]	337 (11; 10–12)	176 (14; 12–16)	161 (9; 8–10)	10.9 (3.5)	4.6 (2.0)	6.4 (2.1)

*High anxiety* Patients with APAIS-A-T > 10, *Low anxiety* Patients with APAIS-A-T < 11, *APAIS* Amsterdam preoperative anxiety and information scale, *APAIS-A-T* APAIS anxiety about anesthesia and surgery score (total APAIS anxiety score), *APAIS-A-An* APAIS anxiety about anesthesia score, *APAIS-A-Su* APAIS anxiety about surgery score. *Data show anxiety scores in all patients. ^a^ Education includes education in school, college and university. ^b^ Surgery of a malignant tumor. ^c^ Surgery involving a burdensome body change. *M* mean, *SD* standard deviation, *95% CI* 95% confidence interval

While approximately half of the patients included in this survey underwent surgery in the departments of Gynecology and Obstetrics (28%), Orthopedic surgery (15%) and General surgery (11%), the remainder of the patients were operated on in another 8 surgical disciplines (Table 3). Surgeries aimed at pain reduction and/or improvement of function (28.9%) were the most common of the 10 different types of surgeries. The numbers of patients belonging to all different types of surgery are shown in Table 4.

**Table 3:** Surgical disciplines

	All patients [n (%; 95% CI)]	High anxiety [n (%; 95% CI)]	Low anxiety [n (%; 95% CI)]	APAIS-A-T [M (SD)]	APAIS-A-An [M (SD)]	APAIS-A-Su [M (SD)]
All patients				9.9 (3.6)	4.3 (1.9)	5.5 (2.1)
Gynecological and obstetric	852 (28; 26–29)	442 (35; 33–38)	410 (22; 20–24)	11.0 (3.6)	4.9 (2.0)	6.1 (2.1)
Orthopedic	471 (15; 14–17)	164 (13; 11–15)	307 (17; 15–19)	9.3 (3.4)	4.1 (1.8)	5.2 (2.0)
General	344 (11; 10–12)	124 (10; 8–12)	220 (12; 11–14)	9.6 (3.1)	4.2 (1.9)	5.4 (2.1)
Trauma	332 (11; 10–12)	118 (9; 8–11)	214 (12; 10–13)	9.2 (3.5)	4.1 (1.8)	5.1 (2.0)
Ears nose and throat	331 (11; 10–12)	116 (9; 8–11)	215 (12; 10–13)	9.5 (3.6)	4.1 (1.9)	5.3 (2.1)
Urological	288 (9; 8–10)	106 (8; 7–10)	182 (10; 9–11)	9.5 (3.4)	4.1 (1.7)	5.4 (2.1)
Ophthalmic	174 (6; 5–7)	66 (5; 4–7)	108 (6; 5–7)	9.3 (3.6)	3.9 (1.8)	5.4 (2.2)
Neurosurgical	137 (4; 4–5)	66 (5; 4–7)	71 (4; 3–5)	10.4 (3.5)	4.2 (1.8)	6.1 (2.2)
Oral and maxillofacial	91 (3; 2–4)	31 (3; 2–4)	60 (3; 3–4)	9.1 (3.3)	3.8 (1.6)	5.3 (2.1)
Cardiac	36 (1; 1–2)	13 (1; 1–2)	23 (1; 1–2)	10.0 (3.6)	4.4 (2.0)	5.5 (2.1)
Dermatological	31 (1; 1–1)	6 (1; 0–1)	25 (1; 1–2)	9.5 (3.1)	4.2 (1.9)	5.3 (1.6)

*High anxiety* Patients with APAIS-A-T > 10. *Low anxiety* Patients with APAIS-A-T < 11. *APAIS* Amsterdam preoperative anxiety and information scale. *APAIS-A-T* APAIS anxiety about anesthesia and surgery score (total APAIS anxiety score). *APAIS-A-An* APAIS anxiety about anesthesia score. *APAIS-A-Su* APAIS anxiety about surgery score. *M* mean. *SD* standard deviation. *95% CI* 95% confidence interval

**Table 4:** Number of patients belonging to the different procedures

Type of procedure	Subgroup	All patients [n (%; 95% CI)]	High anxiety [n (%; 95% CI)]	Low anxiety [n (%; 95% CI)]	APAIS-A-T [M (SD)]	APAIS-A-An [M (SD)]	APAIS-A-Su [M (SD)]
1. Diagnostic procedure	1.1	343 (11; 10–12)	113 (9; 7–11)	230 (12; 11–14)	9.2 (3.35)	4.0 (1.81)	5.1 (1.98)
	1.2	142 (5; 4–5)	76 (6; 5–8)	66 (5; 4–7)	11.0 (3.75)	4.8 (1.89)	6.1 (2.20)
	1.3	332 (11; 10–12)	131 (11; 9–13)	201 (11; 9–12)	9.9 (3.88)	4.5 (1.99)	5.4 (2.25)
2. Imaging and minor interventions	2.1	5 (0; 0–0)	1 (0; 0–0)	4 (0; 0–1)	8.0 (2.74)	3.4 (1.52)	4.6 (2.80)
	2.2	15 (0; 0–1)	3 (0; 0–1)	12 (1; 0–1)	7.4 (3.16)	3.1 (1.31)	4.3 (2.09)
	2.3	8 (0; 0–1)	0 (0; 0–0)	8 (0; 0–1)	7.0 (2.07)	3.3 (1.89)	3.8 (1.58)
	2.4	11 (0; 0–1)	3 (0; 0–1)	8 (0; 0–1)	9.0 (3.41)	4.2 (2.04)	4.8 (1.72)
	2.5	38 (1; 1–2)	17 (1; 1–2)	21 81; 1–2)	9.8 (3.73)	4.3 (1.77)	5.6 (2.26)
3. Trauma	3.1	185 (6; 5–7)	74 (6; 5–8)	111 (6; 5–7)	9.4 (3.51	4.2 (1.84)	5.2 (2.01)
4. Surgery aimed at pain reduction and / or improvement of function	4.1	784 (25; 24–27)	265 (22; 20–25)	459 (24; 22–26)	9.5 (3.47)	4.1 (1.80)	5.3 (2.07)
	4.2	168 (5; 5–6)	63 (5; 4–7)	105 (6; 5–7)	9.3 (3.59)	3.9 (1.82)	5.4 (2.23)
5. Resection (benign)	5.1	501 (16; 15–18)	222 (18; 16–21)	279 (15; 13–17)	10.2 (3.47)	4.6 (1.91)	5.7 (2.00)
6. Resection (malignant)	6.1	323 (11; 9–12)	156 (13; 11–15)	167 (9; 8–10)	10.8 (3.57)	4.5 (1.92)	6.3 (2.09)
7. Highly invasive surgery	7.1	64 (2; 2–3)	31 (3; 2–4)	33 (2; 1–3)	10.7 (3.65)	4.3 (1.90)	6.4 (3.65)
	7.2	22 (1; 0–1)	8 (1; 0–1)	14 (1; 0–1)	10.5 (3.81)	4.6 (1.87)	5.9 (3.81)
8. Cosmetic and reconstructive surgery	8.1	92 (3; 2–4)	33 (3; 2–4)	59 (3; 2–4)	9.6 (3.98)	4.3 (2.13)	5.2 (2.18)
9. Obstetrics	9.1	97 (3; 3–4)	49 (4; 3–5)	48 (3; 2–3)	11.0 (3.62)	5.0 (3.62)	6.0 (1.96)
10. Miscellaneous	10.1	16 (1; 0–1)	6 (1; 0–1)	10 (1; 0–1)	9.3 (2.89)	4.1 (1.73)	5.2 (1.68)

*High anxiety* Patients with APAIS-A-T > 10, *Low anxiety* Patients with APAIS-A-T < 11, *APAIS* Amsterdam preoperative anxiety and information scale, *APAIS-A-T* APAIS anxiety about anesthesia and surgery score (total APAIS anxiety score), *APAIS-A-An* APAIS anxiety about anesthesia score, *APAIS-A-Su* APAIS anxiety about surgery score, *M* mean, *SD* standard deviation, *95% CI* 95% confidence interval

In all patients mean (SD) total preoperative anxiety (APAIS-A-T), anxiety about anesthesia (APAIS-A-An) and anxiety about surgery (APAIS-A-Su) were 9.9 (3.6), 4.3 (1.9), and 5.5 (2.1), respectively. A detailed analysis of prevalence and intensities of patients’ total preoperative anxiety, anesthesia anxiety and surgery anxiety was published recently [2].

### Specific fears

“Anesthesiologist error”, followed by “Waking up during surgery”, and “Not waking up” were the concerns that were rated highest by all patients according to mean mNRS scores. The intensities of all specific anxieties in all patients, in patients with high (APAIS-A-T > 10) and in patients with low anxiety (APAIS-A-T < 11) consistently showed high variability with the SD of each mean mNRS score being almost as high as the mean mNRS score (Table 5). Ranking of these specific anxieties according to their mean mNRS was similar in patients with high and low anxiety. Mean anxiety scores of each specific fear were all significantly higher in patients with high anxiety compared to patients with low anxiety (*p* < 0.0001). The ratio of mean mNRS scores concerning each specific fear in these two groups of patients was consistently at least 2.0 with an average ratio of 2.24 in all specific fears. While “Anesthesiologist error” was the specific fear most patients were affected by (87%; 95% CI 85–88%), “Fatigue and drowsiness” was the fear the least amount of patients felt (74%; 95% CI 73–76%). Ranking of the specific fears according to the fraction of patients affected was almost identical in all patients, patients with high and patients with low anxiety (Table 5).

**Table 5:** Intensities and incidences of specific fears

Specific fears	All patients	High anxiety	Low anxiety	Ratio^a^
Anesthesiologist error	3.9 (3.08)	5.8 (3.01)*	2.7 (2.40)	2.15
	3 [0; 9]	6 [2; 10]	2 [0; 6]	
	87 (85–88)	95 (94–96)	81 (79–82)	
Awareness	3.8 (3.09)	5.6 (3.04)*	2.6 (2.50)	2.15
	3 [0; 9]	5 [1; 10]	2 [0; 6]	
	84 (82–85)	94 (92–95)	77 (75–79)	
Not waking up (death)	3.7 (3.43)	5.9 (3.34)*	2.2 (2.58)	2.68
	3 [0; 10]	6 [1; 10]	1 [0; 6]	
	78 (77–80)	93 (92–95)	68 (66–70)	
Personality changes	3.1 (2.85)	4.6 (2.99)*	2.1 (2.25)	2.19
	2 [0; 8]	4.5 [0; 9]	2 [0; 5]	
	77 (76–79)	89 (88–91)	69 (67–71)	
Nausea and vomiting	3.1 (2.78)	4.4 (2.87)*	2.2 (2.30)	2.00
	2 [0; 7]	4 [0; 8]	2 [0; 5]	
	78 (77–79)	90 (88–91)	70 (68–72)	
Loss of control	2.9 (2.73)	4.6 (2.90)*	1.8 (1.90)	2.56
	2 [0; 7]	5 [0; 9]	1 [0; 4]	
	76 (74–77)	90 (88–91)	67 (64–69)	
Painful measures	2.5 (2.47)	3.6 (2.72)*	1.7 (1.94)	2.12
	2 [0; 6]	3 [0; 8]	1 [0; 4]	
	74 (72–75)	84 (82–86)	66 (64–68)	
Fatigue and drowsiness	2.4 (2.29)	3.5 (2.50)*	1.7 (1.80)	2.06
	2 [0; 6]	3 [0; 7]	1 [0; 4]	
	74 (73–76)	86 (84–88)	66 (64–68)	
All specific fears	3.2 (2.84)	4.7 (2.92)	2.1 (2.21)	2.24

For each specific fear data are mean (SD) (first line), median [10%-; 90%-percentile] of mNRS scores (range 0–10) (second line) and percentage (95% confidence interval) of subjects that rated the specific item using mNRS > 0 (third line). *High anxiety* Patients with APAIS-A-T > 10. *Low anxiety* Patients with APAIS-A-T < 11. * *p* < 0.0001 for comparison of mean anxiety scores of each specific fear in patients with high vs. low anxiety. ^a^ Ratio of mean scores in patients with high versus low anxiety. Items are sorted according to mean mNRS in all patients

Factor analysis of specific fears associated with anesthesia yielded three factors. These factors can be best described as non-physical complications (factor 1), physical complaints (factor 2), and pain (factor 3). Loading of the different specific fears on the different factors is presented in Table 6. The cumulative variance explained by these three factors (factor 1: 31.7%, factor 2: 22.2%, factor 3: 13.6%) was 67.5%. A model with four factors as suggested by the program only would have added 3.1% to the cumulative variance without assigning one of the specific fears to the fourth factor.

**Table 6:** Factor analyses of specific fears associated with anesthesia

Specific fear	Factor 1	Factor 2	Factor 3
Non-physical complications (Factor 1)			
Anesthesiologist error	0.799	0.260	0.253
Not waking up (death)	0.774	0.255	0.191
Awareness	0.657	0.235	0.367
Impairment of personality	0.585	0.430	0.221
Loss of control	0.514	0.334	0.445
Physical complaints (Factor 2)			
Fatigue and drowsiness	0.249	0.924	0.231
Nausea and vomiting	0.355	0.614	0.242
Pain (Factor 3)			
Painful measures	0.269	0.241	0.705

Numbers describe loading of specific fears on factors

### Risk factors for preoperative anxiety

Comparison of 95% confidence intervals of incidences of discrete variables in patients with high versus low anxiety suggests that gender, anesthesia experience, the number of previous surgeries, and surgeries that cause a burdensome change of the body (mutilating surgery) have an impact on total preoperative anxiety (Table 2). The only surgical discipline that had a higher prevalence of patients with high versus low preoperative anxiety was Gynecology and Obstetrics (Table 3). Procedures (Table 4) that had a higher prevalence of patients with high versus low preoperative anxiety included resection of a malignant tumor (group 6) and obstetric procedures (group 9).

### Results from stepwise regression analysis

For all three dependent variables (APAIS-A-T, APAIS-A-An, and APAIS-SU) forward and backward methods for selection of relevant independent variables yielded the same results. Female gender had the strongest impact on all three anxiety dimensions (total preoperative anxiety, anesthesia related anxiety and surgery related anxiety measured by APAIS-A-T, APAIS-A-An and APAIS-A-Su, respectively). While previous negative and positive anesthesia experiences were also shown to be independent predictors of all three anxiety dimensions, other variables (“Highly invasive surgery”, “Mutilating surgery”, “Less than 3 previous surgeries”, “Surgery of a malignant tumor”, “No previous surgery”, “Gynecology and Obstetrics”) were demonstrated to have a significant impact on one or two of these anxiety dimensions only (Table 7). Adjusted r^2^ values of the three models identifying independent predictors of APAIS-A-T, APAIS-A-An, and APAIS-A-Su were 12.6, 11.9, and 10.4%, respectively.

**Table 7:** Independent predictors of preoperative anxiety

Variable	APAIS-A-T	APAIS-A-An	APAIS-A-Su
Female gender	2.02 (12.6)	0.89 (11.2)	0.98 (12.3)
Negative anesthesia experience	1.27 (7.9)	0.75 (9.4)	0.48 (6.0)
Highly invasive surgery^a^	1.07 (6.7)		0.97 (12.1)
Mutilating surgery^b^	0.96 (6.0)		0.64 (8.0)
Less than 3 previous surgeries^c^	0.65 (4.1)	0.46 (5.8)	
Positive anesthesia experience	- 0.63 (3.9)	- 0.38 (4.7)	- 0.29 (3.6)
Surgery of a malignant tumor			0.56 (7.0)
No previous surgery^d^		0.32 (4.0)	
Gynecology and Obstetrics		0.25 (3.1)	

*APAIS* Amsterdam preoperative anxiety and information scale. *APAIS-A-T* APAIS anxiety about anesthesia and surgery score (total APAIS anxiety score). *APAIS-A-An* APAIS anxiety about anesthesia score. *APAIS-A-Su* APAIS anxiety about surgery score. Numbers quantify by how much each anxiety score changes when the variable is present. Numbers in parenthesis describe the magnitude of score change expressed as a percentage of the score range allowing for comparison of effect size of each variable on the three anxiety scores. In variables with a significant effect on more than one anxiety dimension, the order of variables is based on magnitude of score change of overall preoperative anxiety. ^a^ Neurosurgical procedures involving craniotomy and cardiac surgery involving sternotomy. ^b^ Surgical procedures involving a burdensome body change. ^c^ Patients without any and those with 1 or 2 versus those with at least 3 surgeries in their history. ^d^ Patients without any versus those with 1 or 2 and those with more than 2 surgeries in their history

## Discussion

### Specific fears related to anxiety about anesthesia

Results of this study give a detailed picture of the relevance of typical specific fears associated with preoperative anxiety and anesthesia related anxiety in particular by reporting for the first time mean intensities including the amount of variability. The main result in all patients in this context is that the importance which is assigned to all specific fears including the ones with highest ratings is subject to a very large variability reflected by standard deviations almost as big as the mean mNRS. Another main result in this context is that the difference between mean mNRS of items (specific fears) rated highest (“Anesthesiologist error”) and lowest (“Fatigue and drowsiness”) is only 1.5 with an average standard deviation of 2.8 on a scale ranging from 0 to 10. This indicates there is not a single specific fear that is felt most relevant by most patients and that even the specific fears that have the lowest average ratings have a high relevance in many patients. However, despite this big variability, our results indicate a trend identifying specific fears that are likely to be more relevant than others. This assumption concerning the different relevance of the specific fears is supported by another finding of the study. The ranking of percentage of subjects who assigned any degree of concern (i.e. mNRS > 0) to the different items ranging from 87% (“Anesthesiologist error”) to 74% (“Fatigue and drowsiness”) parallels the ranking concerning the importance assigned to the different specific fears according to mean mNRS scores. A comparison of above main results concerning specific fears with results from other studies is extremely hindered by many differences regarding various methodological aspects that could account for the discrepancies between results of past studies and the present study. These include but are not limited to time and place of data collection, choice of specific fears examined, instruments employed to examine and assess incidence and intensity of specific fears, data analysis, and outcome metrics. For instance, Shevde and Panagopoulos [9] who surveyed 800 patients scheduled to undergo surgery used a verbal rating scale / 5-point Likert scale (1 = “not at all”, …, 4 = very much, 5 = extremely) to have subjects rate 20 issues of concern. Results concerning the importance of these 20 issues were only analyzed by ranking the issues according to the relative incidence of subjects assigning “4” or “5” to the issues without presenting relative incidences concerning grades “1” to “3”. While “Anesthesiologist’s qualifications” (45%) and “Experience of anesthesiologist” (43%) were assigned most frequently highest levels of concern (“4” and “5”), “Waking up in the middle of surgery” was only assigned highest level of concern by 24% of subjects. No statistical comparison between the relative incidences of the issues was performed. Similarly important, 95% confidence intervals allowing for roughly assessing differences between reported incidences were not reported either. Taking into account the different underlying statistics and presentation of results concerning the reported ranking of specific fears in their study and the present study, a comparison of study results has to be carried out with great caution. Assuming that “Anesthesiologist’s qualifications” and “Experience of anesthesiologist” in their study compared to “Anesthesiologist error” in the present study can be considered as very similar concerns, results of the present study could be considered to be consistent with Shevde’s and Panagopoulos’ findings. However, while “Being unable to wake up” was rated less frequently (only 37%) as an issue of highest concern compared to “Anesthesiologist’s qualifications” (45%) in their study, “Not waking up” had almost an identical mean intensity compared to “Anesthesiologist error” in the present study. This discrepancy could be accounted for by the fact that “Not waking up” was equated with “death” in the present study (Additional file 3B). This was not the case in the study by Shevde and Panagopoulos. In fact, as explicitly mentioned in the methods section, issues relating to death were purposely excluded to prevent undue patient anxiety. It remains unclear why “Waking up in the middle of surgery” was rated only by 22% as an issue of highest concern with an intermediate ranking similar to “Postop nausea” in their study, whereas “Awareness” had almost an identical mean intensity compared to “Anesthesiologist error” resulting in a second rank in the ranking of all specific fears in the present study. In summary, considering results concerning all issues of concern including the ones that have not been discussed in detail, ranking of the items based on the percentage of patients that reported the different items to be of highest concern in Shevde’s and Panagopoulos’ study parallels the ranking of specific fears based on mean mNRS scores of this study with respect to most of the items studied. However, it is important to emphasize that the present study does not confirm the magnitude of difference between the issues of concern regarding their importance to patients as suggested by Shevde’s and Panagopoulos’ study. While the difference concerning mean intensities of specific fears in this study is small and below the SD of each item, differences in incidences of patients assigning highest concern to the different issues (6–45%) without reporting the amount of variability though, could indicate significant differences between items.

Comparing results of the present study to findings of a study by Matthey and colleagues is even more difficult because of bigger methodological differences [4]. These include above all the instrument used to measure the degree of concern assigned to the 11 items (a three degree verbal rating scale: “not at all concerned”, “somewhat concerned” and “very concerned”) that does not allow to get a differentiated result and also include the way results were reported (incidences of patients assigning the degrees of concern to each specific fear) instead of reporting mean intensities and associated variabilities. Accordingly, items were not ranked according to degree of concern assigned to each specific concern. In addition, there is a limited overlap of items examined in both studies (5 out of 8 items examined in the present study were also examined in their study). Moreover, included subjects and data acquisition also differ significantly (a telephone survey in a non-threatening environment in randomly chosen members of the public in their study vs. a survey using questionnaires in patients waiting for their pre-anesthetic assessment prior to an elective procedure in this study). In light of these limitations, the authors refrain from interpreting results of the present study compared to the results of their study.

To date, only one study has reported on mean scores regarding specific fears associated with preoperative anxiety [5]. The range of mean VAS scores (19–35) is consistent with mean mNRS scores of the present study (2.4–3.9). However, results concerning items that were used in both surveys seem to be inconsistent. While non-physical complications (e.g. “awareness”, “not waking up”) had highest mean scores in the present study, these were rated lowest in the study by Kindler and colleagues. The reasons for these contradictory findings are unknown. However, it is unclear if the mean scores of the items researched in their study were also subject to such a great variability like in the present study because in addition to mean values they only reported rounded SEM (means of all items had a SEM of 1) which makes conversion into exact standard deviations impossible. Accordingly, it is also unclear if items really differ significantly as suggested by the ranking based on mean scores only without showing the associated variability.

Taken together, no definite statement concerning the question to what extent results of this study confirm or refute any of the various diverging findings of past work with regard to the importance of specific fears associated with anesthesia can be made because methodologocal differences between all studies are too great.

Unlike any other study that has been published to date, we also analyzed all specific fears depending on anxiety levels in patients with high (APAIS-A-T > 10) versus low (APAIS-A-T < 11) anxiety. Interestingly, we found that the ranking of the specific fears based on mean mNRS scores was by and large the same in both patient groups and accordingly also compared to the ranking in all patients. In line with these results, we found that the ratio of mean mNRS scores for each specific fear in patients with high versus low anxiety was very similar for most items, with an average of 2.24 and a range from 2.06 to 2.68. Therefore, we conclude that higher preoperative anxiety levels measured by the three APAIS anxiety subscales in patients with high anxiety do not result as we assumed from a heightened level of concern with respect to a few certain specific fears but rather because of a generally higher level of concern regarding most specific fears. Similarly, there are no specific fears that are relatively more important in patients with low anxiety. The only items which had a ratio above average were “not waking up” and “loss of control” suggesting that non-physical complications may play a relatively greater role in patients with high anxiety. It is unclear, however, why other non-physical complications such as “anesthesiologist error” and “awareness” which also belonged to the same dimension of fear according to factor analysis did not have a ratio above average as well.

Factor analysis of specific fears produced three dimensions of specific fears in the present study. Comparison of items loading on the same factor in this study with loading of the same or similar items in previous studies [5, 7, 9] reveals inconsistencies with respect to some of the items. For instance, while “anesthesiologist error” loaded on a factor comprising various “non-physical complications” in the present study, corresponding items (“anesthesiologist’s qualifications” and “experience of anesthesiologist”) loaded on a separate factor called “anesthesiologist’s characteristics” in a study by Shevde and Panagopoulos [9]. Likewise, while “awareness during anesthesia” also loaded on the factor “non-physical complications” in the present study, the same item loaded on a factor including physical complaints such as “nausea and vomiting” and “pain” in a study by Kindler and colleagues [5]. In contrast, item loading was consistent concerning specific fears that loaded on the factor “non-physical complications” in our study and a survey by Mitchell in 460 day-case patients [7]. The reasons for the inconsistencies regarding item loading described above are unclear. However, it is important to realize in this context, that all methodological limitations mentioned above (e.g. differences in data acquisition) that may account for inconsistent results regarding specific fears associated with anesthesia-related anxiety may of course therefore have an impact on factor analysis of these specific fears. Of particular importance in this regard is a great variation in the way factor analysis was performed including the number of and the kind of specific items included as well as the number of dimensions chosen.

### Predictors of preoperative anxiety

Results of this study also give a detailed picture of the associations between several patient variables and total preoperative anxiety including, for the first time, anxiety about anesthesia and anxiety about surgery.

Our findings reconfirm the many previous studies which have identified female gender as a risk factor for preoperative anxiety [5, 14–17, 19–22, 31]. In fact, gender was the variable that had by far the strongest impact on preoperative anxiety, with a similar impact on anesthesia and surgery related anxiety. Interestingly, Caumo and colleagues reported female gender to be a relatively weak risk factor (OR 2.0) compared to “history of cancer” (OR 2.26), “depressive symptoms” (OR 3.22), “minor psychiatric disorders” (OR 5.93), and “history of smoking” (OR 7.47). Results concerning the latter variable are consistent with numerous publications suggesting a relationship between cigarette smoking, anxiety and anxiety disorders (e.g. [32, 33]). However, the magnitude of the reported association is surprising and raises doubts whether the reported effects can be attributed to “smoking” solely. DSM-5 lists increased anxiety as a nicotine withdrawal symptom which is consistent with a large body of literature suggesting an association between anxiety and acute nicotine withdrawal (e.g. [34, 35]). Considering that patients in the study by Caumo and colleagues had to stop smoking during the entire stay in hospital and the unknown timing of data acquisition related to hospital admission it is very likely that nicotine withdrawal played a more important role than “history of smoking” in many patients.

The variable with the second strongest impact on preoperative anxiety was “previous negative anesthesia experience”, thereby confirming results of another study based on univariate analysis [5]. As expected, “previous negative anesthesia experience” had a stronger impact on anesthesia anxiety than on surgery anxiety.

Neurosurgical procedures including craniotomy and cardiac surgery involving sternotomy (i.e. “Highly invasive surgery”) had the third strongest impact on total preoperative anxiety resulting in a rise of the APAIS-A-T score of about 1. This is based on a selective significant impact on surgery related anxiety without a significant impact on anesthesia related anxiety. In contrast, none of the other 9 types of procedures including their subgroups had a significant impact on any of the three APAIS anxiety subscales. The latter findings confirm results of a study by Domar and colleagues who also found no difference in anxiety levels according to multiple regression analysis between patients having minor surgery compared to those having major surgery in 523 participants undergoing a variety of procedures belonging to different surgical disciplines [15]. These results were also consistent with findings by Moerman and colleagues who reported no statistically significant relationship between type of operation and anxiety scores based on univariate analysis [14]. In contrast, Laufenberg-Feldmann and colleagues found that higher grades of surgery was significantly related to higher anxiety levels in patients scheduled to undergo various procedures belonging to different disciplines [19]. These results were consistent with some of the findings of earlier work by Kindler and colleagues. They reported that some operations including thoracic surgery were associated with high preoperative anxiety suggesting that more invasive procedures have a stronger impact on preoperative anxiety [5]. However, this conclusion is inconsistent with another finding by Kindler and colleagues who also reported otorhinolaryngological (ENT) procedures to be associated with high preoperative anxiety. These surprising results were not confirmed by our study including 331 patients scheduled to undergo a wide range of ENT procedures. Results of our study suggest that the association seen by Kindler and colleagues [5] between ENT procedures and high anxiety is likely to be caused by a higher fraction of patients characterized by one or more of the variables shown in the present study to be independently associated with increased total preoperative anxiety (e.g. “female gender” or “previous negative bad anesthesia experience”). Accordingly, we believe that discrepancies between the studies examining the association of surgical procedure with preoperative anxiety [5, 14, 15, 19] can be explained in particular by differences in statistical analysis besides the other methodological differences mentioned above in the context of specific fears. It is also important to remember that to date, there is no uniform, internationally approved classification of invasiveness of surgical procedures. Commonly, classifications of surgical procedures take into account various characteristics such as length of the procedure, organs or tissues involved, blood loss associated with the procedure, length of stay in hospital because of surgery, etcetera. According to these characteristics, surgical procedures are usually classified into two to four groups (grades), e.g. *major* vs. medium vs. minor (e.g. [15, 21, 30]). Considering the various characteristics, it is obvious that the allocation of certain procedures to one of these groups (e.g. *medium* surgery) is subject to a subjective interpretation because each of these characteristics is not well defined either. Moreover, considering that highly invasive procedures were shown to be independent predictors of surgery-related anxiety in the present study, it can be assumed that classifications discriminating between two to four groups only don’t discriminate sufficiently in order to detect differences in invasiveness of procedures and their association to surgery-related anxiety. Taking into account results of all studies examining the association between surgical procedure and the three anxiety dimensions and all their above discussed limitations we conclude that most surgical procedures are no independent predictors of any anxiety dimension. Considering that certain procedures subsumed as “high risk surgeries” were shown to be independent predictors of surgery-related anxiety and thereby also a predictor of preoperative anxiety, it is not unlikely that other high risk surgical procedures (e.g. oesophageal resection involving laparotomy and thoracotomy) could also be independent predictors of these two anxiety dimensions.

Consistent with the evidence related to surgical procedures, none of the surgical disciplines included in the present study were shown to have a significant impact on total preoperative anxiety or surgery related anxiety. This can be easily explained by the fact that each surgical discipline comprises a wide variety of procedures regarding their invasiveness and also concerning other variables shown to be independent predictors of surgery related anxiety (e.g. surgery of a malignant tumor).

Surgical procedures involving a burdensome body change also had a selective significant impact on surgery anxiety resulting likewise in a significant rise in APAIS-A-T score with a similar magnitude to procedures subsumed under “highly invasive surgery”. To date, no other study has examined the association between this variable and preoperative anxiety. However, besides common sense suggesting this variable being a risk factor for anxiety about surgery, it has also been discussed in previous studies when explaining for the association between other variables (e.g. certain surgical procedures) and their association with increased preoperative anxiety [5, 15]**.**

Consistent with previous studies showing that patients without a history of previous anesthesia and / or surgery had significantly higher anxiety levels [5, 16, 22], findings of this study demonstrated that the number of previous surgeries had a significant impact on anesthesia anxiety and thereby on total preoperative anxiety.

History of cancer and cancer surgery are commonly assumed to be associated with higher levels of preoperative anxiety. Interestingly, contradictory results have been published concerning this assumption. While Domar and colleagues found that cancer patients did not have higher anxiety levels [15], Caumo and colleagues [21] reported that history of cancer is a risk factor for preoperative anxiety (OR 2.26) that was even stronger than female gender (OR 2.0). Results of this study reveal that surgery of a malignant tumor is associated with a significant and selective increase in surgery-related anxiety with a similar magnitude to that caused by mutilating surgery. However, unlike surgery involving a burdensome change of the body, cancer surgery does not cause a significant rise in total preoperative anxiety.

To date, the effects of “positive anesthesia experience” on preoperative anxiety have not been studied. As expected, we found this variable to be an independent predictor associated with a drop in all three APAIS anxiety scores. Interestingly, the magnitude of the drop was approximately only half of the magnitude of the rise in all three APAIS anxiety measures associated with “negative anesthesia experience”. This may be due to the negativity bias which is a well-known phenomenon in psychology whereby bad events have a bigger impact on learning processes than good ones [36].

Results of this study demonstrate that age is not an independent predictor of any of the three anxiety dimensions. Past studies have reported inconsistent results in this regard. Above all differences in statistical analyses besides the various other methodological differences mentioned above can most likely account for the disagreeing results of past work. While Kindler and colleagues found patients younger than 37 years compared to patients aged 37–66 years and patients aged > 66 years to have significantly higher STAI scores in males and females using ANOVA [5], Caumo and colleagues also using univariate analysis found the opposite: patients aged 51–60 years compared to patients aged 18–30 years was associated with “high state anxiety” according to STAI scores [21]. However, this result could not be confirmed in the same study using hierarchical multiple conditional logistic regression [21]. There are several limitations associated with these studies. First, It is unclear, what the rationale was to stratify age using 3 [5] or 4 [21] strata chosen in their studies. Unless justified by evidence, any arbitrary stratification of a continuous variable such as age implies the risk of bias. Second, in general, univariate analysis compared to multivariate analysis can be considered less powerful to detect associations between patient variables and the dependent variable (anxiety). Third: Caumo and colleagues used logistic regression to examine this association. However, by constraining the analysis to the “high anxiety” state, associations between patient variables and anxiety cannot be fully examined. Thus, considering all evidence available to date, it can be concluded that age is not an independent predictor of any of the three anxiety dimensions.

Results of the present study reveal that education is not an independent predictor of any of the three anxiety dimensions either. We, therefore confirm results of past work using univariate [5] and multivariate [15, 17] analysis that has suggested education not to be a predictor of preoperative anxiety. In contrast, we refute findings by Caumo and colleagues who reported increasing duration of education to be associated with a higher risk of state anxiety according to multivariate analysis. Surprisingly, they could not show this association using univariate analysis. The main reason for the inconsistencies between the study by Caumo and colleagues and all other studies including the present study could be related to the fact that in their study statistical analysis was constrained to patients with high anxiety. In view of all data available in this respect, we conclude that education is not a predictor of any of the three anxiety dimensions.

Adjusted r2 values of the three models identifying independent predictors of the three anxiety dimensions were low (< 13%). This indicates a poor capability of the identified independent variables to predict the level of any of the three anxiety dimensions. Therefore, the creation of a predictive tool (e.g. a risk score) using these independent variables will always be limited by a large degree of inaccuracy. Accordingly, integration of these independent predictors into every day clinical practice seems to be of limited benefit. Instead, use of instruments such as a mNRS or the APAIS employed in this study to measure the level of anxiety or personal communication with the patient are indispensable to assess each patient’s anxiety level.

### Limitations

This study had several limitations with respect to the design and the conduct of the survey. First, the study was part of a single center survey which reduces generalizability of the results. However, the drawback of this potential bias is outweighed by the very large sample size and by our strict handling of missing data. Second, we intended to focus on specific fears clearly associated with anesthesia. However, a clear assignment of some of the concerns to anesthesia or surgery related anxiety is not always straightforward. Therefore, we did not include all specific concerns that had been studied prior to the start of this survey and that had been shown to be of some relevance (e.g. brain damage [4]). Consequently, we cannot rule out that any of specific fears not included in the present study might be of more relevance than the specific fears that were rated highest in the present study. In addition, given the design of the questionnaire using closed-ended questions and statements that are associated with suggestion and prompting instead of using open-ended questions to elicit patient’s concerns without any suggestive component, we cannot exclude that we were missing important specific fears associated with anesthesia. Third, some variables that were reported by one study to be strongly associated with preoperative anxiety in patients with high levels of preoperative anxiety [21] were not included in this study. This confines the conclusions that can be drawn from the results of the multivariate analysis of associations between the three anxiety dimensions and patient variables examined in the present study. Other limitations that are of relevance to the present study include (a) an uneven distribution of the study subjects among the referring departments, (b) missing documentation of the number of patients declining to participate, and (c) the time of data collection. These have been discussed in detail previously [2].

## Conclusions

Results of this study demonstrate a comprehensive overview of the importance of specific fears typically associated with anesthesia related anxiety in patients from a high income nation. Given the high degree of variance concerning mean scores of all specific fears, the similarity of importance assigned to specific fears by patients with high versus low anxiety, and the low difference in percentages of patients assigning any degree of concern to the different specific fears, we conclude that there is not a single specific fear that is considered important by all or even most patients. Accordingly, patient education materials that aim to cover aspects of patients’ highest concerns should not be constrained to just a few specific fears but should address a wide spectrum of concerns. Results of this study also suggest that a conversation with the patient allowing for an individualized approach compared to generic patient education materials is likely to be far more targeted, and therefore more successful in alleviating patient specific fears. Similarities in the ratios of mean scores of each specific fear in patients with high versus low anxiety indicate it is unlikely beneficial to divide patients in high and low anxiety groups when dealing with specific fears associated with anesthesia related anxiety.

Results of multivariate regression yielded a comprehensive and differentiated picture of which variables were independent predictors of just one, two, or all three anxiety dimensions measured by the three APAIS anxiety scores, how strong their impact was and also clarified inconsistencies reported in previous studies concerning the impact of some variables such as type of surgery, age, and education on total preoperative anxiety [5, 14, 15, 17, 19, 21]. However, the low coefficients of determination of the three models demonstrate that only a small proportion (less than 13%) of the variance for the dependent variables (APAIA-A-T, APAIS-A-An, and APAIS-A-Su) are explained by all variables found to be independent predictors. Therefore, it can be concluded that all three models have a poor predictive capacity to estimate a patient’s level of anxiety and that for every day clinical practice these models have limited use.

## Supplementary information

**Additional file 1.** A German version of the Amsterdam Preoperative Anxiety and Information Scale (APAIS), (Part B1 of the questionnaire). Wording of the German translation of the English version of the APAIS published by Moerman and colleagues [14] and validated by Berth and colleagues [19]. B English version of the Amsterdam Preoperative Anxiety and Information Scale (APAIS). Wording of the English version of the APAIS published by Moerman and colleagues [14]. Items have to be rated by participants on a 1 (not at all) to 5 (extremely) Likert scale.

**Additional file 2.** A Modified numeric rating scale (mNRS) for anxiety assessment- German version. German version of a mNRS used by study participants to rate their level of anesthesia and surgery anxiety. B English translation of Additional file 2A. see Additional file 2A.

**Additional file 3.** A Modified numeric rating scale (mNRS) to assess specific fears - German version. German version of a mNRS used by study participants to rate their level of concern regarding 8 specific fears primarily associated with anesthesia. B English translation of Additional file 3A. see Additional file 3A.

**Additional file 4.** Classification and codification of procedures. Tabular listing showing the allocation of all procedures included in the study to 10 different types of procedures and their subgroups based on the anticipated mental burden of the procedure and the surgical invasiveness.

## Data Availability

The datasets used and analyzed during the current study are available from the corresponding author on reasonable request.

## Abbreviations

APAIS: Amsterdam preoperative anxiety and information scale
APAIS-A-An: APAIS anesthesia related anxiety subscale
APAIS-A-Su: APAIS surgery related anxiety subscale
APAIS-A-T: APAIS total anxiety subscale
APAIS-I: APAIS information subscale
CAPD: Continuous ambulatory peritoneal dialysis
CI: Confidence interval
EBUS: Endobronchial ultrasound
ENT: Ears, nose and throat
ICD: Implantable cardioverter defibrillator
MRI: Magnetic resonance imaging
mNRS: modified numeric rating scale
OR: Odds ratio
RFA: Radiofrequency ablation
SD: Standard deviation
SEM: Standard error of the mean
TACE: Transarterial chemoembolization
VAS: Visual analogue scale
