# Supplementary material for: Preoperative anxiety in adults - a cross-sectional study on specific fears and risk factors
Source: BMC Psychiatry. 2020 Mar 30;20:140. doi: 10.1186/s12888-020-02552-w (PMC7106568; doi:10.1186/s12888-020-02552-w)
Supplement: Supplementary file 1 — Additional file 1. A German version of the Amsterdam Preoperative Anxiety and Information Scale (APAIS), (Part B1 of the questionnaire). Wording of the German translation of the English version of the APAIS published by Moerman and colleagues [14] and validated by Berth and colleagues [19]. B English version of the Amsterdam Preoperative Anxiety and Information Scale (APAIS). Wording of the English version of the APAIS published by Moerman and colleagues [14]. Items have to be rated by participants on a 1 (not at all) to 5 (extremely) Likert scale. [file 12888_2020_2552_MOESM1_ESM.zip › Additional file 1A APAIS GermanR2.docx]

|  | **1**  (gar nicht) | **2**  (wenig) | **3**  (mittel) | **4**  (stark) | **5**  (extrem) |
| --- | --- | --- | --- | --- | --- |
| Ich mache mir Sorgen über die Anästhesie (Narkose) |  |  |  |  |  |
| Die Anästhesie (Narkose) geht mir ständig durch den Kopf |  |  |  |  |  |
| Ich möchte so viel wie möglich über die Anästhesie (Narkose) wissen |  |  |  |  |  |
| Ich mache mir Sorgen über die Operation |  |  |  |  |  |
| Die Operation geht mir ständig durch den Kopf |  |  |  |  |  |
| Ich möchte so viel wie möglich über die Operation wissen |  |  |  |  |  |
