# Supplementary figures and images for: Preoperative anxiety in adults - a cross-sectional study on specific fears and risk factors
Source: BMC Psychiatry. 2020 Mar 30;20:140. doi: 10.1186/s12888-020-02552-w (PMC7106568; doi:10.1186/s12888-020-02552-w)

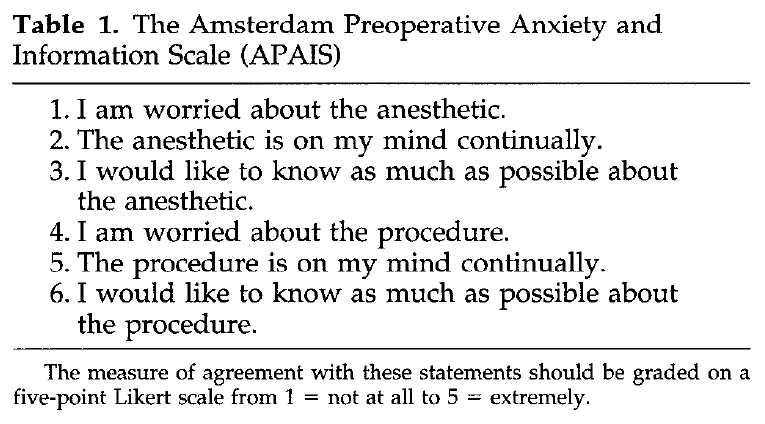

Supplement: Supplementary file 1 — Additional file 1. A German version of the Amsterdam Preoperative Anxiety and Information Scale (APAIS), (Part B1 of the questionnaire). Wording of the German translation of the English version of the APAIS published by Moerman and colleagues [14] and validated by Berth and colleagues [19]. B English version of the Amsterdam Preoperative Anxiety and Information Scale (APAIS). Wording of the English version of the APAIS published by Moerman and colleagues [14]. Items have to be rated by participants on a 1 (not at all) to 5 (extremely) Likert scale. [file 12888_2020_2552_MOESM1_ESM.zip › Additional file 1B APAIS EnglishR2.docx]

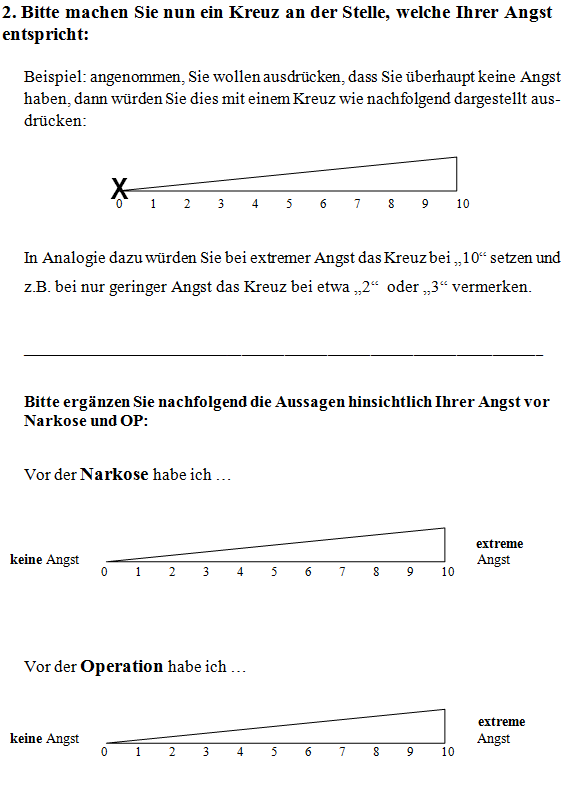

Supplement: Supplementary file 2 — Additional file 2. A Modified numeric rating scale (mNRS) for anxiety assessment- German version. German version of a mNRS used by study participants to rate their level of anesthesia and surgery anxiety. B English translation of Additional file 2A. see Additional file 2A. [file 12888_2020_2552_MOESM2_ESM.zip › Additional file 2A mNRS anxiety GermanR2.docx]
