# Supplementary material for: Preoperative anxiety in adults - a cross-sectional study on specific fears and risk factors
Source: BMC Psychiatry. 2020 Mar 30;20:140. doi: 10.1186/s12888-020-02552-w (PMC7106568; doi:10.1186/s12888-020-02552-w)
Supplement: Supplementary file 2 — Additional file 2. A Modified numeric rating scale (mNRS) for anxiety assessment- German version. German version of a mNRS used by study participants to rate their level of anesthesia and surgery anxiety. B English translation of Additional file 2A. see Additional file 2A. [file 12888_2020_2552_MOESM2_ESM.zip › Additional file 2B English translation of 2AR2.docx]

**2. Please mark a cross at the place which corresponds to your anxiety:**

Example: assuming you want to express that you don’t have any anxiety at all, then you would express this with a cross as shown below:

**X**

| 0 | 1 | 2 | 3 | 4 | 5 | 6 | 7 | 8 | 9 | 10 |
| --- | --- | --- | --- | --- | --- | --- | --- | --- | --- | --- |

By analogy, you would mark the cross in case of extreme anxiety at „10“ and for instance in case of low anxiety at about „2“ or „3“.

_________________________________________________________________________

**Please complete the following statements concerning your anxiety about narcosis and surgery.**

I have …

**no** anxiety **extreme** anxiety

| 0 | 1 | 2 | 3 | 4 | 5 | 6 | 7 | 8 | 9 | 10 |
| --- | --- | --- | --- | --- | --- | --- | --- | --- | --- | --- |

about the **narcosis**

I have

**no** anxiety **extreme** anxiety

| 0 | 1 | 2 | 3 | 4 | 5 | 6 | 7 | 8 | 9 | 10 |
| --- | --- | --- | --- | --- | --- | --- | --- | --- | --- | --- |

about the **operation**
