# Supplementary material for: Preoperative anxiety in adults - a cross-sectional study on specific fears and risk factors
Source: BMC Psychiatry. 2020 Mar 30;20:140. doi: 10.1186/s12888-020-02552-w (PMC7106568; doi:10.1186/s12888-020-02552-w)
Supplement: Supplementary file 3 — Additional file 3. A Modified numeric rating scale (mNRS) to assess specific fears - German version. German version of a mNRS used by study participants to rate their level of concern regarding 8 specific fears primarily associated with anesthesia. B English translation of Additional file 3A. see Additional file 3A. [file 12888_2020_2552_MOESM3_ESM.zip › Additional file 3A mNRS specific fears GermanR2.docx]

**Bitte geben Sie an, worauf sich Ihre Angst vor Narkose begründet:
1.) Angst vor schmerzhaften Maßnahmen (z.B. Legen des Venenzugangs):**

**keine** Angst **extreme** Angst

| 0 | 1 | 2 | 3 | 4 | 5 | 6 | 7 | 8 | 9 | 10 |
| --- | --- | --- | --- | --- | --- | --- | --- | --- | --- | --- |

**2.) Angst vor dem eigenen Kontrollverlust bei Narkose:**

**keine** Angst **extreme** Angst

| 0 | 1 | 2 | 3 | 4 | 5 | 6 | 7 | 8 | 9 | 10 |
| --- | --- | --- | --- | --- | --- | --- | --- | --- | --- | --- |

**3.) Angst vor dem Erwachen bzw. einer „Wachheit“ während der OP:**

**keine** Angst **extreme** Angst

| 0 | 1 | 2 | 3 | 4 | 5 | 6 | 7 | 8 | 9 | 10 |
| --- | --- | --- | --- | --- | --- | --- | --- | --- | --- | --- |

**4.) Angst vor einem Fehler des Narkosearztes:**

**keine** Angst **extreme** Angst

| 0 | 1 | 2 | 3 | 4 | 5 | 6 | 7 | 8 | 9 | 10 |
| --- | --- | --- | --- | --- | --- | --- | --- | --- | --- | --- |

**5.) Angst davor, nicht mehr aufzuwachen (zu sterben):**

**keine** Angst **extreme** Angst

| 0 | 1 | 2 | 3 | 4 | 5 | 6 | 7 | 8 | 9 | 10 |
| --- | --- | --- | --- | --- | --- | --- | --- | --- | --- | --- |

**6.) Angst vor Übelkeit und Erbrechen nach der Narkose:**

**keine** Angst **extreme** Angst

| 0 | 1 | 2 | 3 | 4 | 5 | 6 | 7 | 8 | 9 | 10 |
| --- | --- | --- | --- | --- | --- | --- | --- | --- | --- | --- |

**7.) Angst vor Abgeschlagenheit und langer Müdigkeit nach Narkose:**

**keine** Angst **extreme** Angst

| 0 | 1 | 2 | 3 | 4 | 5 | 6 | 7 | 8 | 9 | 10 |
| --- | --- | --- | --- | --- | --- | --- | --- | --- | --- | --- |

**8.) Angst vor bleibenden Persönlichkeitsbeeinträchtigungen**

**(z.B. Konzentrationsschwäche, Vergesslichkeit o.ä.)**

**keine** Angst **extreme** Angst

| 0 | 1 | 2 | 3 | 4 | 5 | 6 | 7 | 8 | 9 | 10 |
| --- | --- | --- | --- | --- | --- | --- | --- | --- | --- | --- |
