# Supplementary material for: Preoperative anxiety in adults - a cross-sectional study on specific fears and risk factors
Source: BMC Psychiatry. 2020 Mar 30;20:140. doi: 10.1186/s12888-020-02552-w (PMC7106568; doi:10.1186/s12888-020-02552-w)
Supplement: Supplementary file 3 — Additional file 3. A Modified numeric rating scale (mNRS) to assess specific fears - German version. German version of a mNRS used by study participants to rate their level of concern regarding 8 specific fears primarily associated with anesthesia. B English translation of Additional file 3A. see Additional file 3A. [file 12888_2020_2552_MOESM3_ESM.zip › Additional file 3B English translation of 3AR2.docx]

**Please tell us, what your anxiety about narcosis is based on:
1.) Anxiety about painful measures (e.g. placement of a venous catheter):**

**no** anxiety **extreme** anxiety

| 0 | 1 | 2 | 3 | 4 | 5 | 6 | 7 | 8 | 9 | 10 |
| --- | --- | --- | --- | --- | --- | --- | --- | --- | --- | --- |

**2.) Anxiety about loss of control during narcosis:**

**no** anxiety **extreme** anxiety

| 0 | 1 | 2 | 3 | 4 | 5 | 6 | 7 | 8 | 9 | 10 |
| --- | --- | --- | --- | --- | --- | --- | --- | --- | --- | --- |

**3.) Anxiety about waking up and awarenes during surgery, respectively:**

**no** anxiety **extreme** anxiety

| 0 | 1 | 2 | 3 | 4 | 5 | 6 | 7 | 8 | 9 | 10 |
| --- | --- | --- | --- | --- | --- | --- | --- | --- | --- | --- |

**4.) Anxiety about an anesthesiologist error:**

**no** anxiety **extreme** anxiety

| 0 | 1 | 2 | 3 | 4 | 5 | 6 | 7 | 8 | 9 | 10 |
| --- | --- | --- | --- | --- | --- | --- | --- | --- | --- | --- |

**5.) Anxiety about not waking up (dying under anesthesia):**

**no** anxiety **extreme** anxiety

| 0 | 1 | 2 | 3 | 4 | 5 | 6 | 7 | 8 | 9 | 10 |
| --- | --- | --- | --- | --- | --- | --- | --- | --- | --- | --- |

**6.) Anxiety about nausea and vomiting following narcosis:**

**no** anxiety **extreme** anxiety

| 0 | 1 | 2 | 3 | 4 | 5 | 6 | 7 | 8 | 9 | 10 |
| --- | --- | --- | --- | --- | --- | --- | --- | --- | --- | --- |

**7.) Anxiety about fatigue and drowsiness for a long time following narcosis:**

**no** anxiety **extreme** anxiety

| 0 | 1 | 2 | 3 | 4 | 5 | 6 | 7 | 8 | 9 | 10 |
| --- | --- | --- | --- | --- | --- | --- | --- | --- | --- | --- |

**8.) Anxiety about permanent impairment of personality**

**(e.g. lack of concentration, forgetfulness)**

**no** anxiety **extreme** anxiety

| 0 | 1 | 2 | 3 | 4 | 5 | 6 | 7 | 8 | 9 | 10 |
| --- | --- | --- | --- | --- | --- | --- | --- | --- | --- | --- |
