# Supplementary material for: Preoperative anxiety in adults - a cross-sectional study on specific fears and risk factors
Source: BMC Psychiatry. 2020 Mar 30;20:140. doi: 10.1186/s12888-020-02552-w (PMC7106568; doi:10.1186/s12888-020-02552-w)
Supplement: Supplementary file 4 — Additional file 4. Classification and codification of procedures. Tabular listing showing the allocation of all procedures included in the study to 10 different types of procedures and their subgroups based on the anticipated mental burden of the procedure and the surgical invasiveness. [file 12888_2020_2552_MOESM4_ESM.docx]

| **Procedure** | **Codification** |
| --- | --- |
| Abscess incision and drainage (e.g. of a skin or soft tissue abscess) | 4.1 |
| Adenoidectomy | 5.1 |
| Adnexal surgery for benign diseases (e.g. follicular cysts, corpus luteum cysts, endometrioma) | 5.1 |
| Adnectomy for ovarian cancer | 6.1 |
| Amputation (e.g. toe, forefoot, lower leg) | 5.1 |
| Analgesics pump explantation | 10.1 |
| Angiography | 2.1 |
| Angioplasty with /without stenting | 4.1 |
| Anorectal surgery for anal fissures, fistulas and hemorrhoids (e.g. lateral sphincterotomy) | 4.1 |
| Appendectomy | 5.1 |
| Arteriovenous fistula creation (e.g. Cimino fistula) | 4.1 |
| Arthrodesis | 4.1 |
| Arthroscopy (hip, knee, shoulder) | 1.1 |
| Arthroscopic surgery (hip, knee, shoulder), e.g. ACL reconstruction, subacromial decompression | 4.1 |
| Basal cell carcinoma surgery | 6.1 |
| Bimax surgery | 4.1 |
| Biopsy, excisional | 1.3 |
| Brachytherapy procedures (e.g. seed implantation) | 2.2 |
| Breast amputation | 6.1 |
| Breast augmentation surgery | 8.1 |
| Breast conserving surgery | 6.1 |
| Breasts implants, removal | 4.1 |
| Breast implants, replacements | 4.1 |
| Breast reduction surgery | 4.1 |
| Bursal resection | 5.1 |
| Bypass surgery, non-cardiac (e.g. femoropopliteal) | 4.1 |
| CAPD catheter placement | 2.3 |
| Carotid endarterectomy | 4.1 |
| Carpal tunnel surgery | 4.1 |
| Cataract surgery | 4.2 |
| Cavernous angioma resection | 7.1 |
| Central line placement | 2.3 |
| Cesarean section | 9.1 |
| Circumcision | 4.1 |
| Cholecystectomy | 5.1 |
| Chronic subdural hematoma surgery - minimal craniotomy | 7.1 |
| Cochlear implant implantation | 4.1 |
| Coronary artery bypass graft surgery (CABG) | 7.2 |
| Clipping (microsurgical) for cerebral aneurysm | 7.1 |
| Colporrhaphy | 4.1 |
| Crossectomy | 4.1 |
| Curettage | 1.3 |
| Cystectomie | 6.1 |
| Cystoskopy | 1.3 |
| Dacryocystorhinostomy (DCR) | 4.1 |
| Dental treatment / restoration | 4.1 |
| Descemet membrane endothelial keratoplasty (DMEK) | 4.2 |
| Endobronchial ultrasound (EBUS) | 1.3 |
| Endometrial ablation | 1.3 |
| Electroconvulsive therapy (ECT) | 10.1 |
| Epididymectomy | 4.1 |
| Eye exam under general anaesthesia | 10.1 |
| Fasciectomy | 4.1 |
| Forefoot amputation | 4.1 |
| Ganglion cyst excision | 5.1 |
| Gastrectomy | 6.1 |
| Glossectomy (partial, hemi-, and total) | 6.1 |
| Hallux valgus surgery | 4.1 |
| Heart valve surgery (e.g. aortic valve replacement) | 7.2 |
| Hemangioma resection surgery | 5.1 |
| Hematoma drainage | 4.1 |
| Herniated disc surgery | 4.1 |
| Herniotomy (e.g. femoral, incisional, inguinal and umbilical repair) | 4.1 |
| High tibial osteotomy | 4.1 |
| Hydrocele repair | 5.1 |
| Hymenotomy | 4.1 |
| Hysterectomy (abdominal, vaginal) for benign diseases (e.g. adenomyosis, myoma) | 5.1 |
| Hysterectomy (radical; Wertheim Meigs) for malignant diseases | 6.1 |
| Hysteroscopy | 1.3 |
| Ileostoma, reversal of | 4.1 |
| Intravitreal injection | 4.2 |
| Joint replacement surgery (hip, knee, shoulder) | 4.1 |
| Cryotherapy (in e.g. bone tumors) | 2.5 |
| Kyphoplasty | 4.1 |
| Laminectomy | 4.1 |
| Laparoscopy, diagnostic | 1.2 |
| Laparascopic surgery, e.g. cholecystectomy | 5.1 |
| Laparascopic surgery for malignant diseases, e.g. sigmoid colon resection | 6.1 |
| Laparotomy, e.g. Crohn’s surgery (ileocecal resection) | 5.1 |
| Laparotomy for malignant dieseases, e.g gastrectomy | 6.1 |
| Liver resection surgery for benign diseases | 5.1 |
| Liver resection surgery for malignant diseases | 6.1 |
| Lung surgery, thoracotomy | 5.1 or 6.1 |
| Lymphadenectomy | 1.3 |
| Malignant melanoma excicion | 6.1 |
| Marsupialization | 4.1 |
| Mastectomy in males | 8.1 |
| Mastectomy in females | 6.1 |
| Mastoidectomy | 4.1 |
| Maxillary distraction (transverse) | 4.1 |
| Metal removal | 4.1 |
| Meatotomy | 4.1 |
| Microlaryngoscopy | 1.3 |
| Monarc sling operation | 4.1 |
| Meatoplasty | 4.1 |
| Magnetic Resonance Imaging (MRI) | 2.1 |
| Nasal fracture surgery | 4.1 |
| Neck dissection (e.g. radical, modified radical) | 6.1 |
| Nephrectomy | 5.1 or 6.1 |
| Nephrouretectomy | 6.1 |
| Total abdominal hysterectomy | 6.1 |
| Nephrolithotomy, percutaneous | 4.1 |
| Neurofibroma (dermal) removal | 5.1 |
| Nevus resection | 5.1 |
| Nosebleeds surgery | 4.1 |
| Orchiectomy | 5.1 or 6.1 |
| Osteosynthesis, elective | 3.1 |
| Otoplasty | 8.1 |
| Pacemaker implantation | 2.4 |
| Pancreatectomy | 6.1 |
| Panendoscopy | 1.3 |
| Parathyroidektomy | 5.1 or 6.1 |
| Parotidectomy | 5.1 or 6.1 |
| Patellofemoral reconstruction, medial | 4.1 |
| Phlegmon surgery | 4.1 |
| Prostatectomy, radical | 6.1 |
| Prostatectomy, transurethral | 5.1 |
| Radiofrequency ablation | 2.5 |
| Arteriovenous malformation | 4.1 |
| Retinal detachment surgery | 4.2 |
| Resection of metastasis (e.g. liver) | 6.1 |
| Salivary gland duct stenosis or salivary stone surgery | 4.1 |
| Scar revision surgery | 4.1 or 8.1 |
| Sinus surgery, endoscopic | 4.1 |
| Salivary gland resection | 5.1 or 6.1 |
| Spermatocelectomy | 5.1 |
| Mandibulectomy with mandibular reconstruction surgery | 5.1 or 6.1 |
| Vaginectomy (partial, total) | 6.1 |
| Scoliosis surgery | 4.1 |
| Septoplasty and turbinoplasty | 4.1 |
| Skin transplantation | 8.1 |
| Spinal fusion surgery | 4.1 |
| Spinal stenosis surgery | 4.1 |
| Sentinel lymph node dissection | 6.1 |
| Stapedectomy | 4.1 |
| Symblepharolysis | 4.2 |
| Tension free vaginal tape (TVT) placement | 4.1 |
| Thyroidectomy | 5.1 or 6.1 |
| Tonsillectomy | 5.1 |
| Trabeculectomy | 4.2 |
| Transarterial chemoembolization | 2.5 |
| Closure of tracheostomy stoma | 10.1 |
| Transurethral resection o bladder tumor (TURB) | 6.1 |
| Transurethral resection of prostate (TURP) | 5.1 |
| Transvesival prostatectomy | 5.1 |
| Turbinoplasty | 4.1 |
| Tympanoplasty | 41 |
| Urethrotomy, internal (Sachse) | 4.1 |
| Ureteropelvinoplasty | 4.1 |
| Ureteroplasty | 4.1 |
| Uteroplasty | 4.1 |
| Ureterorenoscopy with / without placement of ureteral stent | 1.3 |
| Uterine myoma enucleation | 5.1 |
| Uvulopalatoplasty | 4.1 |
| Vacuum assisted closure | 2.5 |
| Transvaginal, ultrasound guided follicular puncture | 9.1 |
| Vascular bypass surgery | 4.1 |
| Varicocele surgery | 4.1 |
| Varicose veins surgery (ligation and stripping) | 4.1 |
| Vasectomy | 4.1 |
| Video-assisted thoracoscopic surgery (VATS) | 5.1 or 6.1 |
| Vulvectomy | 6.1 |
| Wisdom tooth extraction | 5 |
| Zenker’s diverticulum resection | 4.1 |
